# The Relationship between Mental Health and Music Listening in the Elderly: Is There a Connection?

**DOI:** 10.1192/j.eurpsy.2025.1584

**Published:** 2025-08-26

**Authors:** K. Evangelia, N. Bakalis, S. Kotrotsiou, E. Asimakopoulou

**Affiliations:** 1 Nursing, Frederick University, Nicosia, Cyprus; 2 Nursing, University of Ptras, Patra, Greece

## Abstract

**Introduction:**

All older adults have a strong desire to age healthily and successfully. Because of this, the research community has extensively researched various influences on an older person’s aging experience. One of those influences is music, which is associated with a positive impact on the aging experience and contributes to an individual’s health, wellness, and quality of life.

**Objectives:**

This study aims to explore the relationship between older adults’ mental health and music listening.

**Methods:**

A self-report survey was employed in this study with 168 people over the age of 65, using a convenience sample. The survey had two parts: the first part included socio-demographic information and a variety of music and listening factors, while the second part was a health-related quality of life assessment—the SF-36.

**Results:**

The Mann–Whitney statistical test was used to examine the hypothesis that the mean total score of the SF-36 mental health subscale is different between participants who identified as enjoying music listening and those who did not. Participants who identified as enjoying music listening had a significantly higher overall mental health total score (median = 74.5) than participants who did not enjoy music listening (median = 66.2), U (Enjoy = 38, No Enjoy = 130) = 3037, z = 2.150, p = 0.032.

**Conclusions:**

Music has a positive influence on both mental and physical health in older adults. Listening to music or participating in music-making in late life can positively affect quality of life, particularly for older adults.

**Disclosure of Interest:**

None Declared

